# RNA:DNA triplexes: a mechanism for epigenetic communication between hosts and microbes?

**DOI:** 10.1128/mbio.01982-24

**Published:** 2024-09-19

**Authors:** Holger Bierhoff, Amelia E. Barber, Matthew G. Blango

**Affiliations:** 1 Institute of Biochemistry and Biophysics, Center for Molecular Biomedicine (CMB), Friedrich Schiller University, Jena, Germany; 2 Institute of Microbiology, Friedrich Schiller University, Jena, Germany; 3 Cluster of Excellence Balance of the Microverse, Friedrich Schiller University, Jena, Germany; 4 Leibniz Institute for Natural Product Research and Infection Biology—Hans Knöll Institute ((Leibniz-HKI), Jena, Germany; Karlsruhe Institute of Technology (KIT), Karlsruhe, Germany

**Keywords:** microbial pathogenesis, RNA:DNA triple helix, epigenetics, fungi, microbial communication

## Abstract

Molecular communication between host and microbe is mediated by the transfer of many different classes of macromolecules. Recently, the trafficking of RNA molecules between organisms has gained prominence as an efficient way to manipulate gene expression via RNA interference (RNAi). Here, we posit a new epigenetic control mechanism based on triple helix (triplex) structures comprising nucleic acids from both host and microbe. Indeed, RNA:DNA triplexes are known to regulate gene expression in humans, but it is unknown whether interkingdom triplexes are formed either to manipulate host processes during pathogenesis or as a host defense response. We hypothesize that a fraction of the extracellular RNAs commonly released by microbes (e.g., bacteria, fungi, and protists) and their hosts form triplexes with the genome of the other species, thereby impacting chromatin conformation and gene expression. We invite the field to consider interkingdom triplexes as unexplored weaponry in the arms race between host and microbe.

## OPINION/HYPOTHESIS

## MICROBES MANIPULATE THEIR HOSTS

The manipulation of host organisms by microbial pathogens and symbionts is well-documented. The parasitic protozoan *Toxoplasma gondii* inhibits the fear response of infected rodents towards feline predators, leading to increased predation and parasite dissemination (1); gram-negative bacteria of the genus *Wolbachia* are nutritional mutualists of filarial worms and reproductive parasites of arthropods, where they skew the sex of offspring to promote reliance on infection, alongside other complex phenotypes (2); and the entomopathogenic fungus *Ophiocordyceps unilateralis* infects ants and controls their behavior to promote fungal dispersal to the remainder of the colony (3). These are just a few striking examples that underscore how host–microbe interactions manifest in multifarious ways. The microbes in these and myriad other examples rely on a variety of mechanisms to exert these effects, including secreted or injected proteins, chemical mediators, and even the intercellular trafficking of RNA molecules associated with extracellular vesicles (EVs).

The best-characterized mechanisms of manipulation are likely the protein effectors described in bacterial pathogens and plant pathogenic fungi. Bacteria like *Yersinia pestis*, *Salmonella enterica*, *Pseudomonas syringae*, and *Xanthomonas* spp. all rely on a battery of proteins to control host gene expression and immunity (4), similar to fungal and oomycete plant pathogens (e.g., *Magnaporthe oryzae* and *Phytophthora* spp.) that harbor a variety of effector molecules to facilitate virulence (5). Protein effectors are accompanied by a tremendous diversity of other metabolites and macromolecules, including RNA molecules that are only starting to be appreciated with advances in sequencing technology and improved computational strategies. The majority of these interactions rely on the transfer of non-coding RNAs (ncRNA), which have been classically studied for their endogenous role within the cell, but in more recent studies have been highlighted for their importance in microbial communication, virulence, and even host immunity.

## EXTRACELLULAR RNA IS RELEASED BY HOSTS AND MICROBES TO INFLUENCE INTERACTIONS

Across all domains of life, ncRNAs serve as versatile regulators of gene expression, acting at both the transcriptomic and genomic levels. It is increasingly appreciated that many of the same ncRNAs that function endogenously within a cell are also secreted with moonlighting functions in intercellular or even interkingdom communication (6). Both free and extracellular vesicle-associated RNAs have recently attracted attention as novel mediators of intercellular communication in eukaryotes and prokaryotes alike [reviewed in (6–8)]. EVs not only enable the export of RNA but also facilitate the delivery, protection, targeting, and trans-kingdom exchange of these and other biomolecules, for example, between microbes and their hosts. The importance of RNA molecules as effectors is best illustrated by the small ncRNAs transferred to plant hosts by fungal pathogens like *Botrytis cinerea* to facilitate infection, which have been reviewed extensively elsewhere (9, 10).

The ncRNA are key regulators of gene expression and impact many aspects of microbial pathogenesis and symbiosis (11–13). Small ncRNAs (<200 nucleotides), including the prominent microRNAs and short-interfering RNAs, silence gene expression via RNAi, and have even been linked to epigenetic regulation in higher eukaryotes (14, 15) . Notably, transfer of small ncRNAs between hosts and pathogens is used for cross-kingdom RNAi, which relies on RNA silencing to skew the outcome of infection (16). Studies have also reported that tRNA and rRNA fragments are transported in EVs between fungal pathogens and plants (17), further increasing the diversity of RNAs with a potential for cross-kingdom regulation. Incredibly, intact mRNAs were recently shown to be transported from plant host to fungal pathogen, where they are translated into functional proteins that reduce virulence (18).

**Fig 1:**
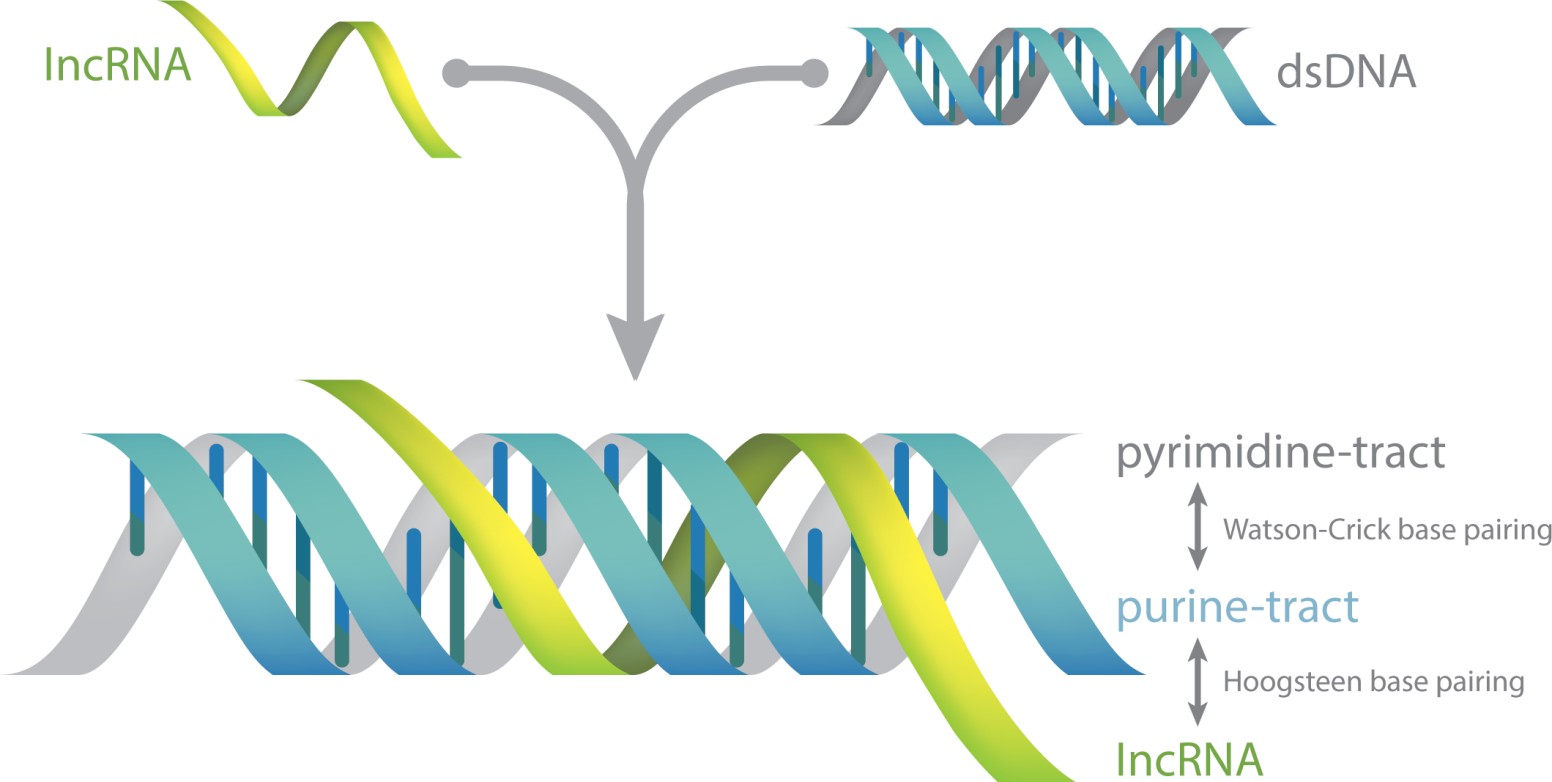
RNA:DNA triplex structure. Single-stranded RNA molecules of appropriate sequence composition, complementarity, and length can be accommodated in the major groove of double-stranded DNA, forming triplex structures via Hoogsteen base pairing with the purine-rich tract.

Long ncRNAs (lncRNAs; >200 nucleotides) are a highly heterogeneous class of RNAs that include transcripts with both housekeeping and gene regulatory functions. Regarding the latter, many lncRNAs impact gene transcription via recruitment of chromatin modifiers in *cis* or in *trans*, thereby altering epigenetic signatures at distinct genomic loci (19). One mechanism by which lncRNAs and even small ncRNAs like microRNA interact directly with DNA is by the sequence-specific engagement into RNA:DNA triple helices (triplexes). In these triple-stranded nucleic acid structures, a stretch of approximately 12 to 30 nucleotides of the RNA is accommodated in the major groove of double-stranded DNA and forms Hoogsteen base pairs with the purine-rich strand (Fig. 1) (20–22). Bioinformatic and experimental evidence indicates that RNA:DNA triplex formation is a widespread phenomenon in eukaryotes (23), although the contribution of triplex formation to microbial pathogenesis and immune response remains almost completely unexplored. The majority of the described interactions occur between endogenous RNA sequences and the corresponding genomic DNA, although one example of triplex formation between host microRNAs and a genome-integrated provirus exists, suggesting the potential for a more complex host–pathogen interplay (24).

## RNA:DNA TRIPLEXES AS A MEANS OF INTERKINGDOM COMMUNICATION

Given that triplex structures can form in *trans* and have relaxed sequence specificity for RNA:DNA pairing, they are well-suited for epigenetic cross talk between hosts and microbes. Moreover, endogenous triplexes control gene expression during immunity as interferon-responsive elements that can act as triplex-forming sites to inhibit gene expression (25). Endogenous host microRNAs antagonize lentiviral replication by forming triplexes with the provirus (24), suggesting that these intriguing structures may have more important functions in immunity. We go a step further and postulate that heterologous ncRNAs transferred between hosts and microbes can form triplexes in the genome of the recipient organism. There is a knowledge gap regarding the existence and role of such interkingdom RNA:DNA triplexes during pathogenesis; however, in the case of viral infections, it has been demonstrated that the influenza A virus triggers the formation of endogenous triplexes in the host, while human cells use microRNA:provirus triplexes to fight lentiviral replication (25, 26).

**Fig 2:**
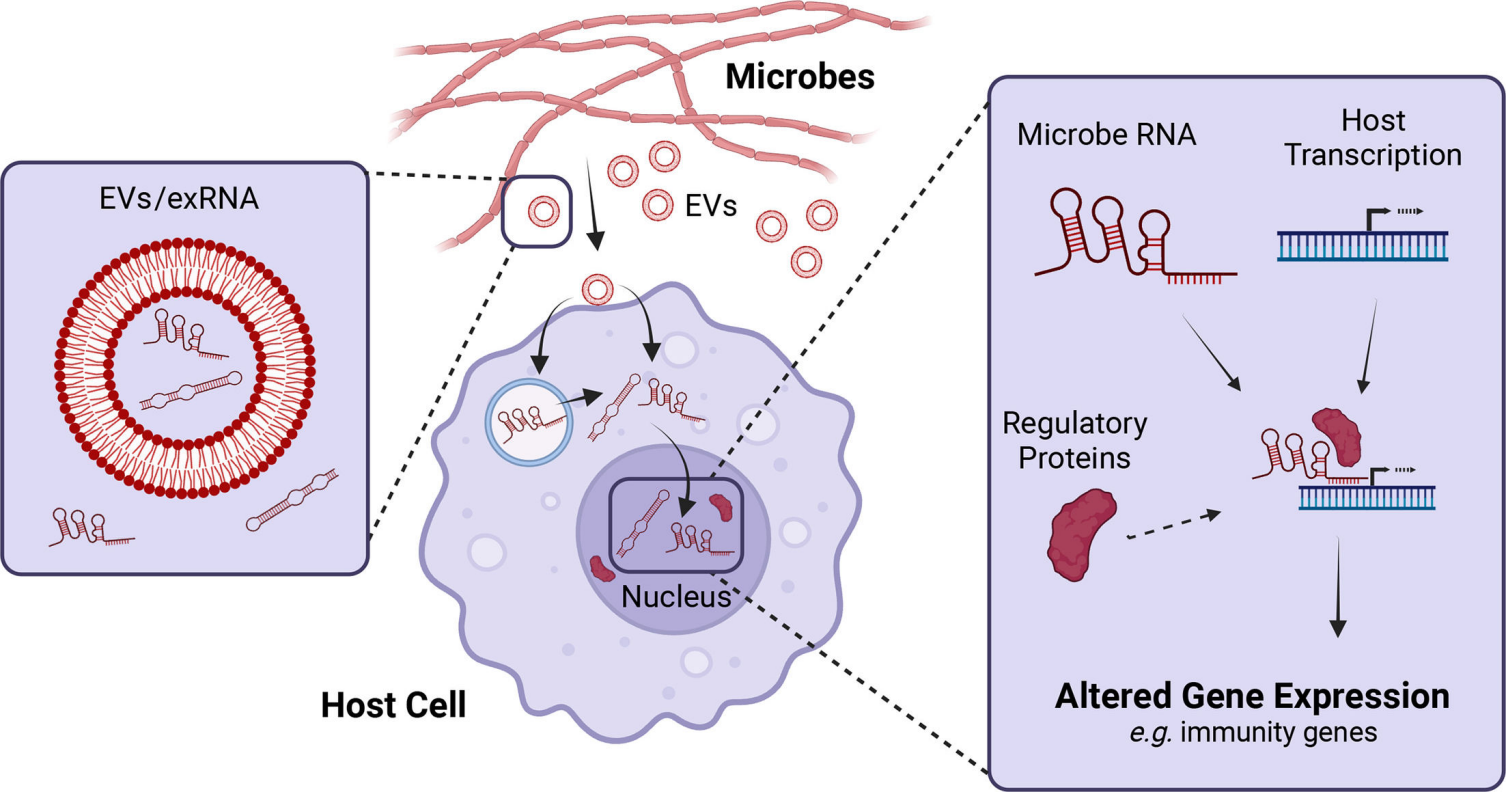
Model of putative interkingdom triplex regulation. A proposed model in which microbe-derived extracellular RNA is delivered to host cells in association with extracellular vesicles (EVs). Upon internalization, the RNA is trafficked to the nucleus to form interkingdom triplex structures capable of facilitating epigenetic gene regulatory changes, potentially through recruitment of host or microbe proteins to the target genomic DNA. Created with BioRender.com.

Here, we aim to extend this concept by proposing that secreted microbial RNAs interact with host (e.g., human, plant, and insect) genomic DNA to form interkingdom RNA:DNA triplexes during infection or even symbiosis (Fig. 2). Of note, because of the compelling evidence of endogenous RNA:DNA triplexes in humans, they are the prime candidates for also harboring interkingdom triplexes. However, triplexes are also conceivable in other cross-species settings, for instance between bacterial, fungal, and/or protist partners in microbial communities, highlighting the broad applicability and potential impact of such interactions. The formation of these triplex structures would likely facilitate recruitment of additional protein modulators and allow for the control of host gene expression, potentially by altering the epigenome of the target organism to enforce long-term changes. It seems probable that host organisms also transfer ncRNAs to pathogens to attenuate virulence via interkingdom triplex structures. Determining the specific contexts and consequences of RNA:DNA triplexes will be exciting to unravel in the coming years.

The most obvious place to look for such triplexes might be within eukaryotic fungal pathogens, due to their complex transcriptomes, previously defined interkingdom RNA signaling pathways, and abundant encoding of both short and long ncRNAs. In this case, we posit that fungal pathogens could use this mechanism to gain epigenetic control over host defense genes and to acquire an advantage during infection, as just one example. A compelling piece of evidence in support of this hypothesis is that the many fungal pathogens, like nearly all microbes examined, secrete RNA in association with EVs during their lifecycle (27). These nanometer-sized, membrane-bound bodies are well-characterized to carry a variety of macromolecular cargo, including ncRNAs of varying lengths. Upon entry into a host cell, fungal-derived small ncRNAs would exert the previously described RNAi in the cytoplasm, while lncRNAs would traffic to the nucleus to Hoogsteen base pair with their target sites and change the epigenetic configuration. In this way, a two-pronged manipulation of gene expression would be achieved. In addition to fungi, it is entirely plausible that bacteria and protozoa also manipulate host organisms in a similar manner. To date, only a few examples exist of small RNAs trafficked from bacteria to their host organisms, with a particularly compelling example being the transfer of *Legionella pneumophila* small RNAs to the host as mimics of host microRNAs to regulate immunity (28). The large number of bacterial small RNAs linked to stress response and virulence may indicate that new functionalities in triplex formation remain to be discovered.

## WHAT CAN WE LEARN FROM INTERKINGDOM RNA:DNA TRIPLEXES?

The field of RNA:DNA triplexes is in its infancy, and understanding the rules of triplex formation beyond Hoogsteen base pairing is a central, yet unresolved question. The identification and characterization of interkingdom triplexes will likely uncover new biochemical features required for triplex formation, and thus will aid in fully appreciating these regulatory structures. Additionally, these studies will significantly expand our knowledge of the regulation of the host immune system, establishing the importance of RNA:DNA triplexes for control of the immune response to pathogens in a variety of different systems. We suspect that interkingdom triplexes represent a new layer in the molecular arms race between pathogens or symbionts and their hosts. Therefore, we are excited to learn how RNA:DNA triplexes contribute to the immune response and how pathogens coopt, manipulate, or even disrupt these structures for their own benefit.

The idea that microbial RNAs form triplex structures poses the challenge of delivering these RNA molecules into the nucleus of a eukaryotic organism, where the genomic DNA resides. The literature suggests that RNA is readily taken up during host pathogenesis to reach the cytoplasm where it can induce RNAi (29, 30), although the mechanisms are largely unknown (9). Longer RNAs are also capable of making this journey from one organism to the cytoplasm of another, as recently demonstrated by the transfer of host plant mRNAs to fungal pathogens for translation (18). These results suggest that extracellular RNA species have somehow solved the endosomal escape problem that is still a bottleneck in RNA therapeutics delivery in many cases. However, to form triplexes, the heterologous RNAs must also overcome the nuclear membrane, perhaps relying on specific RNA structures, RNA modifications, or binding to protein transporters. Research on interkingdom triplexes will help to elucidate these features, thus uncovering novel subcellular trafficking mechanisms that may further inform RNA therapeutics approaches and widen the impact of such studies. At the level of extracellular RNA exchange, we anticipate that new therapeutic targets will also be discovered. With the advent of RNA-based therapeutics, we are only beginning to understand the potential targets available for the treatment of devastating infections like those caused by fungal pathogens. Our treatment options are quite limited here, but RNA-based therapeutics show promise in combatting these challenging infections (10, 31).

It is worth noting that RNA:DNA triplexes do not form in isolation in the cell, instead we suspect that a wide variety of host and microbe proteins are likely to associate with such structures and influence functional outcomes. The field of RNA-guided DNA-binding proteins is a growing one, and most readers already know the clear example of the RNA-guided DNA endonuclease Cas9 (32); however, other RNA-guided DNA binding factors exist. For example, RNA-guided transcription factors like the TnpB-like nuclease-dead repressors (TldRs) are leveraged by bacteriophages to control host organism gene expression in a manner similar to that of CRISPR interference (33). Since the identification of Cas9, a variety of other RNA-guided DNA endonucleases have also been described, including a eukaryotic variation called Fanzor that functions similarly (34). Ultimately, we expected that studies of RNA:DNA triplex biology will unearth new examples of RNA-guided enzymes important for microbial communication and infection biology, by providing a new perspective for discovery.

We challenge the field to re-evaluate existing data sets and explore the potential for interkingdom triplexes, taking advantage of the rapidly advancing computational and experimental toolkits surrounding the study of RNA triplex biology (16–18). Although previously difficult to assess, recent advances in bioinformatic predictions and molecular tools are opening the door to a more robust description of these fascinating, non-canonical nucleic acid structures. For example, triplex prediction tools like Triplexator (35) or TriplexAligner (23) could be used to interrogate potential triplex formation between the well-annotated human genome and non-coding RNAs released by protozoan parasite *Leishmania* (36) or uropathogenic *E. coli* in the bladder (37). Alternatively, the excellent data sets collected by our colleagues in the plant community using fungal and oomycete pathogens could be revisited to search for RNA:DNA triplex-forming potential there (38, 39). Ultimately, we are excited to see our hypothesis tested and learn more about how RNA is breaking through the boundaries between the kingdoms of life.
